# *Staphylococcus aureus* from Subclinical Cases of Mastitis in Dairy Cattle in Poland, What Are They Hiding? Antibiotic Resistance and Virulence Profile

**DOI:** 10.3390/pathogens11121404

**Published:** 2022-11-23

**Authors:** Edyta Kaczorek-Łukowska, Joanna Małaczewska, Patrycja Sowińska, Marta Szymańska, Ewelina Agnieszka Wójcik, Andrzej Krzysztof Siwicki

**Affiliations:** 1 Department of Microbiology and Clinical Immunology, Faculty of Veterinary Medicine, University of Warmia and Mazury in Olsztyn, Oczapowskiego 13, 10-719 Olsztyn, Poland; 2 Proteon Pharmaceuticals, Tylna 3a, 90-364 Łódź, Poland

**Keywords:** AMR, biofilm, *mastitis*, *S. aureus*, virulence factors

## Abstract

Bovine mastitis is a common disease worldwide, and staphylococci are one of the most important etiological factors of this disease. *Staphylococcus aureus* show adaptability to new conditions, by which monitoring their virulence and antibiotic resistance mechanisms is extremely important, as it can lead to the development of new therapies and prevention programs. In this study, we analyzed *Staphylococcus aureus* (*n* = 28) obtained from dairy cattle with subclinical mastitis in Poland. The sensitivity of the isolated strains to antibiotics were confirmed by the disc diffusion method. Additionally, minimum inhibitory concentration values were determined for vancomycin, cefoxitin and oxacillin. Genotyping was performed by two methods: PCR melting profile and MLVF-PCR (multiple-locus variable-number tandem-repeat fingerprinting). Furthermore, the presence of antibiotic resistance and virulence genes were checked using PCR reactions. The analyzed strains showed the greatest resistance to penicillin (57%), oxytetracycline (25%) and tetracycline (18%). Among the analyzed staphylococci, the presence of 9 of 15 selected virulence-related genes was confirmed, of which the *icaD*, *clfB* and *sea* genes were confirmed in all staphylococci. Biofilm was observed in the great majority of the analyzed bacteria (at least 70%). In the case of genotyping among the analyzed staphylococci (combined analysis of results from two methods), 14 patterns were distinguished, of which type 2 was the dominant one (*n* = 10). This study provides new data that highlights the importance of the dominance of biofilm over antibiotic resistance among the analyzed strains.

## 1. Introduction

Mastitis can be caused by more than 200 aetiological factors, of which one of the more frequently mentioned is *Staphylococcus aureus*. This bacterium is classified as a contagious pathogen, which means that it spreads in the herd due to improper hygiene on the farm. This pathogen can cause chronic infections that are difficult to cure with conventional therapy and, as a result, lead to enormous economic losses in the dairy industry [1]. *S. aureus* usually causes subclinical mastitis, which is very difficult to treat (due to ahigh seeding rate of infected animals and chronic recurrent infections). Probably, this is related to the numerous virulence factors present in this pathogen, such as the ability to produce biofilm, the production of toxins, or various enzymes designed to damage and better occupy the infected area [2,3]. On the other hand, it is related to the occurrence of antibiotic resistance among these bacteria, which has arisen from the overuse of these active substances in veterinary medicine and in agriculture [1,3,4]. Especially dangerous are methicillin-resistant staphylococci strains, which pose a direct threat to humans (through food-borne pathogens), and these, unfortunately, are observed more frequently each year in veterinary medicine [4]. Although the problem of *S. aureus* in dairy cattle has been known for years, a perfect preventive therapy or treatment has not yet been developed. This is due to the rapid genetic variability of this pathogen and the lack of knowledge about the interaction between the bacterium and the host [5,6]. Studies from around the world have shown that there is no clear pattern in the distribution of virulence genes (adhesins or toxins) among bovine isolates. Depending on the area from which the strains originate, a different antibiotic resistance profile can be observed. Such a situation significantly complicates the results of mastitis prevention research and the development of new therapies, and further research towards a better understanding of these bacteria should be conducted. Only through this will it finally be possible to develop methods for inhibiting the development of these pathogens. Hence, the aim of our study was to examine what mechanisms of virulence and antibiotic resistance are dominant in *S. aureus* isolated in northeastern Poland among dairy cattle with subclinical mastitis.

## 2. Materials and Methods

### 2.1. Milk Samples

All milk samples were collected from dairy cattle with subclinical mastitis from various farms located in northeast Poland (Warmia and Mazury voivodeship) between 2016 and 2019. To standardize the study group, only staphylococci from subclinical cases were selected for the study. Subclinical milk samples were included in the study if there were no clinical signs and the somatic cell count (SCC) was higher than 400,000 cells/mL.

### 2.2. Bacteriological Identification

Milk samples (0.01 mL) for bacteriological examination were transferred with a calibrated inoculation loop onto Columbia agar medium supplemented with 5% defibrinated sheep blood (Oxoid, Basingstoke, UK) and Chapman medium (Oxoid, Basingstoke, UK). The plates were incubated at 37 °C under aerobic conditions for 48 h. The grown isolates were subjected to microbiological analysis, which included evaluation of the morphology of bacterial colonies, Gram staining, and selected biochemical tests (tests for catalase, coagulase and selected latex tests (Staphytect Plus) (Oxoid, Basingstoke, UK)). The final identification of *S. aureus* was confirmed by PCR of the *nuc* and *femA* genes [7] (primers in Supplementary Table S1). In total, 28 *S. aureus* strains were included in this study. To avoid testing epidemiologically related isolates, only one isolate per dairy farm was included.

### 2.3. Antimicrobial Susceptibility Testing—Disc Diffusion Method

The sensitivity of the isolated strains to antibiotics were confirmed by the disc diffusion method [8] with 21 antimicrobials commonly used in Poland for veterinary treatment: amoxicillin + clavulanic acid (20 + 10 µg) (AMC), enrofloxacin (5 µg) (ENR), clindamycin (2 µg) (CL), tetracycline (30 µg) (TE), erythromycin (15 µg) (E), ceftriaxone (30 µg) (CEQ), cloxacillin (5 µg) (OB), neomycin (30 µg) (N), penicillin G (10U) (P), marbofloxacin (MAR), ceftiofur (30 µg) (EFT), bacitracin (10 µg) (B), cefamandole (30 µg) (MA), cefoperazone (75 µg) (CFP), gentamycin (10 µg) (CN), oxytetracycline (30 µg) (OT), penicillin/novobiocin (40 µg) (PNV), trimethoprim/sulfamethoxazole 1:19 (25 µg) (SXT), ubrolexin (CFX), rifampicin (5 µg) (RD) and cefapirin (30 μg) (CPR). All discs were purchased from Oxoid (Basingstoke, UK). The resistance to antibiotics was assessed according to the CLSI—Clinical and Laboratory Standards Institute [9] guidelines using the quality control strain *Staphylococcus aureus* ATCC 25923. All strains were categorized as sensitive (S), intermediate (I) or resistant (R) to the tested active substances.

### 2.4. Antimicrobial Susceptibility Testing—E-Test, Minimum Inhibitory Concentration (MIC)

MIC testing for oxacillin, cefoxitin and vancomycin was carried out using ready-made e-test strips (Oxoid, Basingstoke, UK) according to the manufacturer’s instructions. Mueller–Hinton BBL II agar supplemented with 2% NaCl (*w*/*v*) was used for oxacillin, while Muelle–Hinton agar without NaCl was used for the other two substances. The plates were incubated at 35 °C+/−2 °C for 18 h. Interpretation was performed according to CLSI guidelines [9].

### 2.5. Biofilm Formation

This experiment was performed using polystyrene microtiter plates with flat bottoms based on the techniques described by Ebrahimi et al. [10] with slight modification. All strains (*n* = 28) were incubated in TSB with the addition of 1% glucose under aerobic conditions at 37 °C. The results were read after 24, 48 and 72 h using an ELISA plate reader (Sunrise absorbance reader, Tecan, Austria). Each strain was analyzed in 8 wells in triplicate. Biofilm production was interpreted according to the criteria described by Stepanović et al. [11]. The mean optical density (OD) of the negative control + 3 standard deviations of the negative control was considered the cut-off (ODc = 0.188), and biofilm producers were therefore categorized as follows:

Not a biofilm producer: OD ≤ ODc (all strains with OD values below 0.188),
Weak biofilm producer: ODc < OD ≤ 2  ×  ODc (all strains with OD values above 0.188 and below 0.375),
Moderate biofilm producer: 2 × ODc  < OD ≤ 4  ×  ODc (all strains with OD values above 0.375 and below 0.752),
Strong biofilm producer: OD > 4  ×  ODc (all strains with OD values above 0.752).

### 2.6. DNA Isolation

Bacterial DNA was extracted using an ExtractMe DNA bacteria kit (Blirt, Gdańsk, Poland) according to the manufacturer’s recommendations. Eluted DNA concentrations and quality were measured using a BioSpectrometer (Eppendorf, Hamburg, Germany) and stored at −20 °C for further analysis.

### 2.7. PCR MP (PCR Melting Profile)

The PCR MP procedure, which was based on the digestion of genomic DNA with restriction enzymes and the ligation of the obtained DNA restriction fragments with an oligonucleotide adaptor, followed by PCR amplification with a reduction in the denaturation temperature during each cycle, was optimized for *Staphylococcus* spp. In this study, approximately 0.5 μg of DNA (25 μL) was digested with HindIII (Thermo Scientific, Waltham, MA, USA). Following incubation at 37 °C for 30 min and inactivation at 65 °C for 15 min, the ligation mix was added: 4 μL of adapter (two oligonucleotides), 2.8 μL of ligation buffer (Thermo Scientific), 0.4 μL T4 DNA ligase (5 U/L, Thermo Scientific, MA, USA) and 0.5 µL 25 mM ATP. The samples were incubated at 16 °C for 1 h and then heated in a thermoblock at 65 °C for 10 min. Afterwards, the samples were cooled at room temperature for 10 min. The PCR was carried out in a 25 μL reaction mixture containing 14.75 μL PCR grade water, 4 μL ligation product, 2.5 μL 10× PCR buffer (Shark, Blirt, Gdańsk, Poland), 1 μL 50 mM MgCl_2_, 2 μL of a deoxynucleoside triphosphate (dNTP) mixture, 0.5 μL (2 U) of polymerase (Hypernova Blirt, Gdańsk, Poland) and 0.25 μL of primer AD-P (CTCACTCTCAACAACGTCGACAGCTT (5′→3′). The denaturation temperature was determined during the optimization of two *S. aureus* strains using a gradient thermal cycler (Eppendorf Mastercycler Nexus, Hamburg, Germany) with a gradient range of 78–88 °C for the denaturation step. The PCRs were performed as follows: (i) 7 min at 72 °C—initial denaturation, releasing of unligated nucleotides; (ii) 90 s at a 78–88 °C gradient across the thermal block—denaturation, (iii) 24 cycles of denaturation at 60 s at 78–88 °C gradient across the thermal block, annealing at 72 °C for 2 min and elongation at 72 °C for 2 min 15 s, with a final 72 °C for 5 min after the last cycle. MP-PCRs for all isolates were performed as described above using the established optimal denaturation temperature of 80 °C. Each PCR product (8 μL) was run on a 1.5% agarose gel, and the amplification patterns were determined by examination on Simply Safe (EurX, Gdańsk, Poland) stained gels illuminated by UV light (Alpha Innotech, Fc8800, Markaryd, Sweden). The amplicon sizes were determined by comparing the bands with a 100-bp DNA mass ladder (Fermentas, Waltham, MA, USA).

### 2.8. MLVF-PCR

The MLVF (multiple-locus variable-number tandem-repeat fingerprinting) typing procedure and a set of 5 primers for the genes *ClfA1*, *ClfB1*, *SdrCDE*, *SpaI and SspA1* were analogous to the previously published scheme for *S. aureus* differentiation [12]. 

The PCR was carried out in a 50 μL reaction mixture containing 25 μL of 2 × PCR TaqNova-RED, 0.5 µM each of primers *ClfB1-F*, *ClfB1-R*, *SpaI-F*, and *SpaI-R*, 1 µM each of primers *ClfA1-F*, *ClfA1-R*, *SdrCDE-F*, and *SdrCDE1-R*, 2 µM each of primers *SspA1-F* and SspA1-R, and 5 ng of template DNA. DNA fragments were amplified on a thermocycler (Eppendorf Mastercycler Nexus, Hamburg, Germany) using the following cycling conditions: (i) 30 s at 98 °C—initial denaturation, and (ii) 20 cycles of denaturation for 10 s at 98 °C, annealing at 60 °C for 30 s and elongation at 72 °C for 30 s, with a final extension at 72 °C for 5 min after the last cycle. Each PCR product (8 μL) was run on a 1.5% agarose gel, and the amplification patterns were determined by examination on Simply Safe (EurX, Gdańsk, Poland) stained gels illuminated by UV light (Alpha Innotech, Fc8800, Markaryd, Sweden). The amplicon sizes were determined by comparing the bands with a 100-bp DNA mass ladder (Fermentas, MA, USA).

### 2.9. PCR Detection of Antimicrobial Resistance and Virulence Genes of Staphylococcus aureus

The resistance genes for macrolides (*erm*(A), *erm*(B) and *erm*(C)), tetracyclines (*tet*(K), *tet*(L) and *tet*(M)), aminoglycosides (*aad-6* and *aphA-3′)*, beta-lactams (*blaZ* and *mecA)*, sulfonamides (*sul*)and antiseptic resistance genes (*gac* and *smr*) were assessed by PCR. All genes were chosen in accordance with the available literature. In the case of virulence, the investigated genes were *sea*, *seo*, *sen*, *lukM*, *lukd*, *clfA, icaA*, *icaC*, *icaB*, *icaD*, *clfB*, *sdrC*, *eno*, *bap* and *etb*. Primer sequences, product sizes and annealing temperatures are summarized in Supplementary Table S1 [13,14,15,16,17,18,19,20,21,22] and Table S2 [23,24,25,26,27,28,29]. All primers were synthesized by Genomed S.A. (Warsaw, Poland). Amplification reactions were carried out with a HotStarTaq Plus Master Mix Kit (Qiagen, Hilden, Germany) in a Nexus Gradient thermocycler (Eppendorf, Hamburg, Germany). The 20-μL reaction sample contained 10 μL of HotStarTaq Plus Master Mix 2×, 1 μL of primers (final concentration 0.4 μM), 2 μL of 10× CoralLoad Concentrate (Qiagen), 4 μL of RNase-free water, and 2 μL of DNA. Cycling conditions were as follows: 95 °C for 5 min, followed by 35 cycles of 94 °C for 30 s, annealing temperature for 30 s, and 72 °C for 60 s, with a final extension of 72 °C for 10 min. Ten microlitres of PCR product were electrophoresed on 2% agarose gel in the presence of Midori Green Advance (Nippon Genetics, Düren, Germany) at 120 V for 60 min. The results were read using the Quantum ST5 Gel Documentation System (Vilber, Eberhardzell, Germany). To confirm the specificity of the amplicons obtained, some PCR products of interest were randomly chosen and purified using a CleanUp kit (A&A Biotechnology, Gdynia, Poland) for sequencing (Genomed S.A., Warsaw, MA, Poland).

## 3. Results

### 3.1. Antimicrobial Susceptibility Testing—Phenotypic and Genotypic Assessments

The analyzed *S. aureus* samples showed the greatest resistance to penicillin (57%), oxytetracycline (25%) and tetracycline (18%). In contrast, they showed intermediate susceptibility to penicillin with novobiocin (75%), oxytetracycline (45%) and enrofloxacin (50%). The analyzed strains were relatively susceptible to the rest of the tested antibiotics (between 85% and 100% of the isolates were susceptible). Among the bacteria analyzed, no methicillin- or vancomycin-resistant strains were confirmed (Table 1). Of the 15 antibiotic resistance-related genes analyzed in the studied staphylococci, only two, *tet(K)* (64%) and *blaZ* (82%), were confirmed.

### 3.2. Detection of Virulence Genes by PCR

Among the strains analyzed, 9 of the 15 selected virulence-related genes were confirmed (Table 2). The *icaD*, *clfB* and *sea* genes were confirmed in all staphylococci. In 87% of the analyzed strains, the presence of the *lukM*, *lukD* and *sdrC* genes was confirmed, while only 3% of the *S. aureus* strains showed the presence of the *sea* and *sen* genes.

### 3.3. Biofilm Formation

Among the analyzed strains, the majority showed the ability to produce biofilms (at least 70% of the strains at each analyzed time). In the case of our study, it was observed that the number of strains capable of biofilm production and its intensification increased as the incubation time of the biofilm plates increased (Table 2).

### 3.4. Genotyping

Among the staphylococci analyzed, nine patterns were obtained using the PCR MP method, and ten patterns were obtained using the MLFV method. By using a combined analysis of the results from these two methods, it was possible to distinguish 14 patterns, of which type 2 was the dominant pattern (*n* = 10). Unfortunately, for one strain of analyzed *S. aureus* (ID-228), no MP PCR pattern was obtained. For all pointed isolates, the PCR MP and MLVF reactions were performed in triplicate to confirm the obtained results (Table 2, Figures S1 and S2).

## 4. Discussion

The introduction of prevention programs against contagious pathogens that cause mastitis in dairy cattle has resulted in a decrease in the aetiology of mastitis, and this phenomenon was also observed in the area that we analyzed [30]. Despite intensive hygienic and therapeutic efforts, *S. aureus* has not been completely eliminated. When infection occurs in the herd as a consequence of numerous virulence and antibiotic resistance mechanisms, this pathogen is very difficult to completely eliminate, and worse, recurrences are often observed, making staphylococcus a challenge for both veterinary doctors and dairy cow owners [31,32].

In the case of the *S. aureus* that we analyzed, the presence of high antibiotic resistance at both phenotypic and genotypic levels was not observed. As Arturrson et al. [2] reported, cows with subclinical mastitis caused by these staphylococci are often removed from the herd after lactation. To the best of our knowledge, such a technique is also practiced in our country, which may be the reason why antibiotic resistance is so low. Both the antibiograms and the presence of genes showed only increased resistance to tetracyclines (*tet(K)* 64%) and beta-lactams (*blaZ* 82%). These results are consistent with studies conducted in Brazil, Norway and China, where phenotypic resistance to tetracycline and penicillin was also observed [33,34]. Interestingly, in the case of research conducted by Martini et al. [33], the most common genes among the analyzed staphylococci were also found to be *blaZ* (97%) and *tet(K)* (84%). Our results suggest that both penicillin and tetracycline may have been overused in the past in the area we analyzed, making it necessary to consider whether they should be withdrawn from use in staphylococcal mastitis infections. Such a measure could prevent the spread of antibiotic resistance to tetracyclines and penicillin to other bacterial species (horizontal gene transfer).

Observing the emergence of strains of methicillin-resistant *S. aureus* (MRSA) and vancomycin-resistant *S. aureus* (VRSA) is extremely important. Initially, these strains were associated mainly with the hospital environment; for years, they have also been observed in the veterinary environment [35,36]. Among the staphylococci we analyzed, no methicillin- or vancomycin-resistant strains were confirmed. Further monitoring of this phenomenon is necessary, as the emergence of such strains among animals used for food purposes poses a direct threat to humans (food-borne pathogens).

High susceptibility to substances other than penicillins and tetracyclines is, in our opinion, a positive phenomenon that offers great opportunities in antibiotic therapy for veterinarians. Nevertheless, it should be mentioned here that high sensitivity in vitro does not guarantee therapeutic success [37]. It is estimated that in the case of infections caused by *S. aureus*, the chances of real or apparent cure vary from 4% to 92%, depending on factors such as the characteristics of the pathogen, the condition of the animals, herd management or treatment regimen [32,38]. Therefore, further work on developing replacement or supplementary therapies for antibiotic therapy should be carried out in the context of these bacteria.

It is well known that staphylococci have the capacity for genetic variation and adaptation to attack new species. Clones of *S. aureus* that are currently able to attack the udder in cattle most likely originate from clones of human origin, and this transformation has occurred gradually since the Neolithic age after the domestication of these animals, among others. To invade a new animal species, the bacterium must acquire or lose its virulence mechanisms so that it can survive anatomically and physiologically in the new location [5]. Therefore, monitoring the virulence mechanisms of staphylococci according to the infected animal species and selecting/developing prevention and treatment programs based on this is extremely important. In our study, the predominant virulence mechanism at both the phenotypic and genotypic levels was the ability of the tested strains to produce biofilms. In the case of the phenotypic study, at all three analyzed incubation times (24, 48 and 72 h), the majority of the staphylococci showed the ability to produce biofilms (above 70%). Among the biofilm-related genes, the presence of only two genes, *eno* and *icaD*, was confirmed, but interestingly, they were present in all the strains tested (including those that did not show the ability to produce biofilm). These results are partially consistent with the observations of other authors, in which some of the more frequently observed genes were *icaD* and *eno*, and the presence of these genes was not dependent on the ability to produce biofilms in vitro [39,40]. Numerous publications have shown that even if at a given moment the bacterium does not use these genes for biofilm production, in the future they may start using them due to various factors, e.g., change in temperature or time, or contact with some substance/surface [39,41,42]. In light of this information, the appearance of these genes in the strains we analyzed is already a worrisome fact, and the monitoring of this phenomenon should continue. In the case of the bacteria we analyzed, the presence of both the *icaA* and *bap* genes was not observed. These observations are consistent with the results of Gajewska et al. [40], who also analyzed *S. aureus* strains from dairy cattle but from a different region of Poland. In their study, the *icaA* gene was also not observed, and the *bap* gene was present in only 25% of the analyzed strains, despite the ability to produce biofilm in in vitro conditions. In our opinion, this may indicate that these genes do not play a key role in the ability to produce biofilms in the area we analyzed. As reported by Chen et al. [39], although the *bap* protein was first linked to the ability of staphylococci to produce biofilms, the gene encoding this protein is located on the *SPIbov2* pathogenicity island, which is found in only a few *S. aureus* strains of bovine origin. Perhaps such strains have not occurred thus far in the area under analysis.

Additionally, it should be mentioned here that the number of nonbiofilm-forming strains decreased with the increasing incubation time. These results are consistent with those obtained by Oliverira et al. [43], in which, in addition to the demonstration of a large number of staphylococci showing the ability to produce this structure, a correlation between the result and reading time was also observed. We agree with the suggestion of those authors that different strains may have different times of biofilm production and that interpreting the results only after 24 h can significantly affect the results.

For the strains we analyzed, we used two genotyping methods to check the relatedness of the *S. aureus* strains isolated from dairy cattle in northeastern Poland. For the strains we analyzed, the combined method distinguished 14 types, of which 35.7% were classified as type 2. These results suggest moderate genetic variability in the area we analyzed, which is good information in the context of developing targeted preventive measures or new therapies. According to Kasela et al. [44], the use of two genotyping techniques simultaneously yields more accurate and reliable typing results for the staphylococci analyzed. This can be seen in our results, in which using only one method, 9 types (with the MP PCR method) or 10 types (with the MLFV method) were distinguished, while 14 types were distinguished using a combined analysis of the two techniques. That kind of approach seems to be reasonable solution in case of staphylococci, which are quite clonal bacteria. In that case, the use of pulsed-field gel electrophoresis (PFGE), considered to be the gold standard for bacteria differentiation, does not allow for tracing epidemiological links between *Staphylococcus* strains [45] as its discriminatory power is not high enough.

As mentioned earlier, staphylococci demonstrate the ability to adapt to new conditions. Therefore, monitoring their mechanisms of virulence and antibiotic resistance is extremely important, as it allows us to complete our knowledge of the pathogenesis of this pathogen and thus may contribute to the development of new more effective therapies and prevention programs in the future. In summary, in the area we analyzed, the staphylococcal isolates showed lower antibiotic resistance than virulence. Among the virulence factors, biofilm formation was dominant, and new prevention programs should be focused on this, especially since it not only affects the virulence of a given bacterium but also decreases the effectiveness of applied active substances used for the treatment of cattle.

## Figures and Tables

**Table 1:** Antibiotic resistance results for *Staphylococcus aureus* strains analyzed (*n* = 28). Legend: amoxicillin + clavulanic acid 20 + 10 µg (AMC), enrofloxacin 5 µg (ENR), clindamycin 2 µg (CL), tetracycline 30 µg (TE), erythromycin 15 µg (E), ceftriaxone 30 µg (CEQ), cloxacillin 5 µg (OB), neomycin 30 µg (N), penicillin G 10U (P), marbofloxacin (MAR), ceftiofur 30 µg (EFT), bacitracin 10 µg (B), cefamandole 30 µg (MA), cefoperazone 75 µg (CFP), gentamycin 10 µg (CN), oxytetracycline 30 µg (OT), penicillin/novobiocin 40 µg (PNV), trimethoprim/Sulfamethoxazole 1:19 25 µg (SXT), ubrolexin (CFX), rifampicin 5 µg (RD), Cefapirin 30 µg (CPR), oxacillin (OX), cefoxitin (FOX), vancomycin (VAN), ND: not detected, red (R): resistance, orange (I): intermediate, green: sensitive, ND: not detected.

STRAIN ID/ANTIMICROBIAL	AMC	ENR	CL	TE	OT	E	CEQ	OB	N	P	PNV	MAR	SXT	EFT	CN	CFP	MA	B	RD	CPR	VAN	FOX	OX	CONFIRMED ANTIBIOTIC RESISTANCE GENES
287																								ND
494																								tet(K), blaZ
522																								tet(K), blaZ
294																								blaZ
292																								blaZ
476																								tet(K), blaZ
342																								tet(K), blaZ
510																								tet(K), blaZ
321																								tet(K), blaZ
165																								blaZ
398																								blaZ
322																								tet(K), blaZ
377																								tet(K), blaZ
536																								tet(K), blaZ
312																								tet(K), blaZ
360																								blaZ
399																								tet(K), blaZ
493																								tet(K), blaZ
535																								tet(K), blaZ
390																								tet(K), blaZ
509																								blaZ
495																								tet(K), blaZ
227																								blaZ
397																								blaZ
556																								tet(K), blaZ
545																								blaZ
544																								tet(K), blaZ
228																								tet(K), blaZ
R%	0	0	0	18	25	0	0	0	0	57	7	0	0	0	0	0	0	0	0	0	0	0	0	
I%	0	50	4	0	46	7	14	0	0	0	75	4	0	4	0	7	0	0	11	0	0	0	0	

**Table 2:** Biofilm production capacity of *Staphylococcus aureus* (*n* = 28) from subclinical mastitis samples from the northeast region of Poland after 24, 48 and 72 h with virulence pattern and PCR MP and MLVF profile. Color green: no biofilm, orange: weak biofilm, blue: medium biofilm, red: strong biofilm, ND: no profile in PCR MP and MLVF analysis.

Strain ID	24	48	72	CONFIRMED VIRULENCE GENES	MP PCR	MLVF	Combined
287				*lukM*, *lukD*, *clfA*, *icaD*, *clfB*, *sdrC*, *eno*	4	1	1
494				*lukM*, *lukD*, *clfA*, *icaD*, *clfB*, *sdrC eno*	4	2	2
522				*lukM*, *lukD*, *clfA*, *icaD*, *clfB*, *sdrC*, *eno*	4	1	1
294				*lukM*, *clfA*, *icaD*, *clfB*, *sdrC*, *eno*	4	6	3
292				*lukD*, *clfA*, *icaD*, *clfB*, *sdrC*, *eno*	4	1	1
476				*lukM*, *lukD*, *clfA*, *icaD*, *clfB*, *sdrC eno*	4	1	1
342				*lukM*, *lukD*, *clfA*, *icaD*, *clfB*, *sdrC*, *eno*	2	2	4
510				*lukM*, *lukD*, *clfA*, *icaD*, *clfB*, *sdrC*, *eno*	4	2	2
321				*lukM*, *lukD*, *clfA*, *icaD*, *clfB*, *sdrC*, *eno*	4	2	2
165				*lukM*, *lukD*, *clfA*, *icaD*, *clfB*, *sdrC*, *eno*	4	7	5
398				*lukM*, *lukD*, *clfA*, *icaD*, *clfB*, *sdrC*, *eno*	4	2	2
322				*lukM*, *lukD*, *clfA*, *icaD*, *clfB*, *sdrC*, *eno*	4	2	2
377				*lukM*, *lukD*, *clfA*, *icaD*, *clfB*, *sdrC*, *eno*	1	1	6
536				*lukM*, *lukD*, *clfA*, *icaD*, *clfB*, *sdrC*, *sea*, *seo*, *eno*	7	9	7
312				*lukM*, *lukD*, *clfA*, *icaD*, *clfB*, *sdrC*, *eno*	4	3	8
360				*lukM*, *lukD*, *clfA*, *icaD*, *clfB*, *eno*	2	1	9
399				*lukM*, *lukD*, *clfA*, *icaD*, *clfB*, *sdrC*, *eno*	4	2	2
493				*lukM*, *lukD*, *clfA*, *icaD*, *clfB*, *eno*	4	2	2
535				*seo*, *sen*, *LukM*, *lukD*, *clfA*, *icaD*, *clfB*, *eno*	7	9	7
390				*lukM*, *lukD*, *clfA*, *icaD*, *clfB*, *sdrC*, *eno*	4	4	10
509				*lukM*, *lukD*, *clfA*, *icaD*, *clfB*, *sdrC*, *eno*	4	2	2
495				*lukM*, *lukD*, *clfA*, *icaD*, *clfB*, *sdrC*, *eno*	4	2	2
227				*lukM*, *lukD*, *clfA*, *icaD*, *clfB*, *sdrC*, *eno*	5	5	11
397				*lukM*, *lukD*, *clfA*, *icaD*, *clfB*, *sdrC*, *eno*	4	2	2
556				*lukM*, *lukD*, *clfA*, *icaD*, *clfB*, *sdrC*, *eno*	9	2	12
545				*lukM*, *lukD*, *clfA*, *icaD*, *clfB*, *sdrC*, *eno*	4	1	1
544				*clfA*, *icaD*, *clfB*, *sdrC*, *eno*	8	10	13
228				*lukM*, *lukD*, *clfA*, *icaD*, *clfB*, *sdrC*, *eno*	ND	8	14
no biofilm *n* [%]	9 (30)	4 (13)	3 (10)	Number of pattern types	9	10	14
				Number of strains without a profile	1	0	0
weak biofilm	16 (53)	17 (57)	13 (43)				
moderate biofilm	4 (13)	5 (17)	11 (37)				
strong biofilm	1 (3)	3 (10)	3 (10)				

## Data Availability

The datasets generated are available from the corresponding author on reasonable request.

## Supplementary Materials

The following supporting information can be downloaded at: https://www.mdpi.com/article/10.3390/pathogens11121404/s1. Table S1. Primer sets used for the identification of *Staphylococcus aureus*, and antimicrobial resistance determinants in staphylococci isolated from subclinical cases of bovine mastitis in Poland; Table S2. Primer sets used biofilm production and other virulence factors in *Staphylococcus aureus* isolated from subclinical cases of bovine mastitis in Poland. Figure S1. MP-PCR HindIII profiles of *Staphylococcus* genomes. Figure S2. MLVF-PCR profiles of *Staphylococcus* genomes: marker 100bp, S. aureus_058PP2016-084PP2016, S. aureus_089PP2016-091PP2016, S. CNS_002PP201.
