# Supplementary material for: Staphylococcus aureus from Subclinical Cases of Mastitis in Dairy Cattle in Poland, What Are They Hiding? Antibiotic Resistance and Virulence Profile
Source: Pathogens. 2022 Nov 23;11(12):1404. doi: 10.3390/pathogens11121404 (PMC9781172; doi:10.3390/pathogens11121404)
Supplement: Supplementary file 1 [file pathogens-11-01404-s001.zip › pathogens-2019369-supplementary-done.pdf]

pathogens

MDPI

Table S1. Primer sets used for the identification of *Staphylococcus aureus*, and antimicrobial resistance determinants in staphylococci isolated from subclinical cases of bovine mastitis in Poland.

Primer function	target gene	Primer sequence (5′–3′)	Amplicon size (bp)	Annealing temperature (°C)	Reference
identyfication	*femA*	F:CTTACTTACTGGCTGTACCTG R:ATGTCGCTTGTTATGTGC	686	43	[7]
	nuc	F:TCGCTTGCTATGATTGTGG R:GCCAATGTTCTACCATAGC	359	46	
macrolides	*erm(A)*	F: TCTAAAAAGCATGTAAAAGAAA R:CGATACTTTTTGTAGTCCTTC	533	52	[13]
	erm(B)	F: GAAAAGTACTCAACCAAATA R: AGTAACGGTACTTAAATTGTTTA	639	41	[14]
	erm(C)	F: ATCTTTGAAATCGGCTCAGG R: CAAACCCGTATTCCACGATT	295	47	[15]
tetracyclines	*tet(K)*	F: TCGATAGGAACAGCAGTA R: CAGCAGATCCTACTCCTT	169	44	[16]
	tet(L)	F: TCGTTAGCGTGCTGTCATTC R: GTATCCCACCAATGTAGCCG	267	50	
	tet(M)	F: GTGGACAAAGGTACAACGAG R:CGGTAAAGTTCGTCACACAC	406	50	
aminoglycosides	*aad-6*	F: AGAAGATGTAATAATATAG R: CTGTAATCACTGTTCCCGCCT	978	37	[17]
	aphA-3′	F: GGGGTACCTTTAAATACTGTAG R: TCTGGATCCTAAAACAATTCATCC	848	50	[18]
beta-lactam	*blaZ*	F: AAGAGATTTGCCTATGCTTC R: GCTTGACCACTTTTATCAGC	517	45	[19]
	mecA	F: GTAGAAATGACTGAACGTCCGATAA R:CCAATTCCACATTGTTTCGGTCTAA	310	52	[20]
sulfonamides	*sul1*	F: TTTCCTGACCCTGCGCTCTAT R:GTGCGGACGTAGTCAGCGCCA	793	52	[21]
antiseptic resistance genes	*qac*	F: ATGCCTTATATTTATTTAATAATAGCC R: ATGCGATGTTCCGAAAATGTTTAAC	321	47	[22]
	smr	F: CTATGGCAATAGGAGATATGGTGT R:CCACTACAGATTCTTCAGCTACATG	417	52	

Table S2. Primer sets used biofilm production and other virulence factors in *Staphylococcus aureus* isolated from subclinical cases of bovine mastitis in Poland.

	Primer function	target gene	Primer sequence (5′–3′)	Amplicon size (bp)	Annealing temperature (°C)	Reference
enterotixins	Enterotoxin A	*sea*	F: AAAGTCCCGATCAATTTATGGCTA R: GTAATTAACCGAAGGTTCTGTAGA	210	55	[23]
	Enterotoxin O	*seo*	F:AAATGATTCTTTATGCTCCG R: AAAGCACATTGTCATGGTGA	300	42	[24]
	Enterotoxin N	*sen*	F: ATGAGATTGTTCTACATAGCTGCAAT R: AACTCT GCTCCC ACTGAA C	680	48	
Leukotoxin bi-component pore-forming complexes	Leukotoxin LukD	*lukD*	F: CTTATCAGGTGGATTGAATG R: CTATACTCCAGGATTAGTTTCT	526	44	
	Leukocidin LukM	*lukM*	F: AACGTGTTTTAATAGCGTCATC R: CACTTCTTACTAATGCTGGGTA	792	46	
Surface Factor-clumping factor	Surface Factor- clumping factor	*clfA*	F: GGCTTCAGTGCTTGTAGG R: TTTTCAGGGTCAATATAAGC	1042	43	[25
	Surface Factor- clumping factor	*clfB*	F: TGTCGAATAAGCAGAATAAG R: GGTGATGATTGTGGTAAATC	505	43	[24]
	Surface Factor- Bone sialoprotein-binding protein	*sdrC*	F: CAACATTGATTTTTGGGTTAAGTGG R: GCAAATGCGACTTGTTCAAA	802	45	
biofilm	Intercellular adhesion protein A	*icaA*	F: ACAGTCGCTACGAAAAGAAA R: GGAAATGCCATAATGACAAC	103	45	[26]

	intercellular adhesion	*icaB*	F: CTGATCAA-<br>GAATTTAAATCACAAA<br>R: AAAGTCCCATAAGCCTGTTT	302	45	
	intercellular adhesion	*icaC*	F: TAACTTTAGGCGCATATGTTTT<br>R: TTCCAGTTAGGCTGGTATTG	400	45	
	intercellular adhesion	*icaD*	F: ATGGTCAAGCCCAGA-<br>CAGAG<br>R: CGTGTTTTCAACATTTAATGCAA	198	42	[27]
	laminin binding protein	*eno*	F: ACGTGCAGCAGCTGACT<br>R: CAACAGCATCTTCAGTACCTTC	302	55	[28]
	biofilm associated protein	*bap*	F: CCCTATATCGAAGGTG-<br>TAGAATTG<br>R: GCTGTTGAAGTTAATACTGTACCTGC	971	60	[29]
Protease	Exfoliative toxin B	*etB*	F: TTACAAGCAAAAGAATACAGCG<br>R: GGAAGATTATGTTGTCCGCC	641	47	[24]

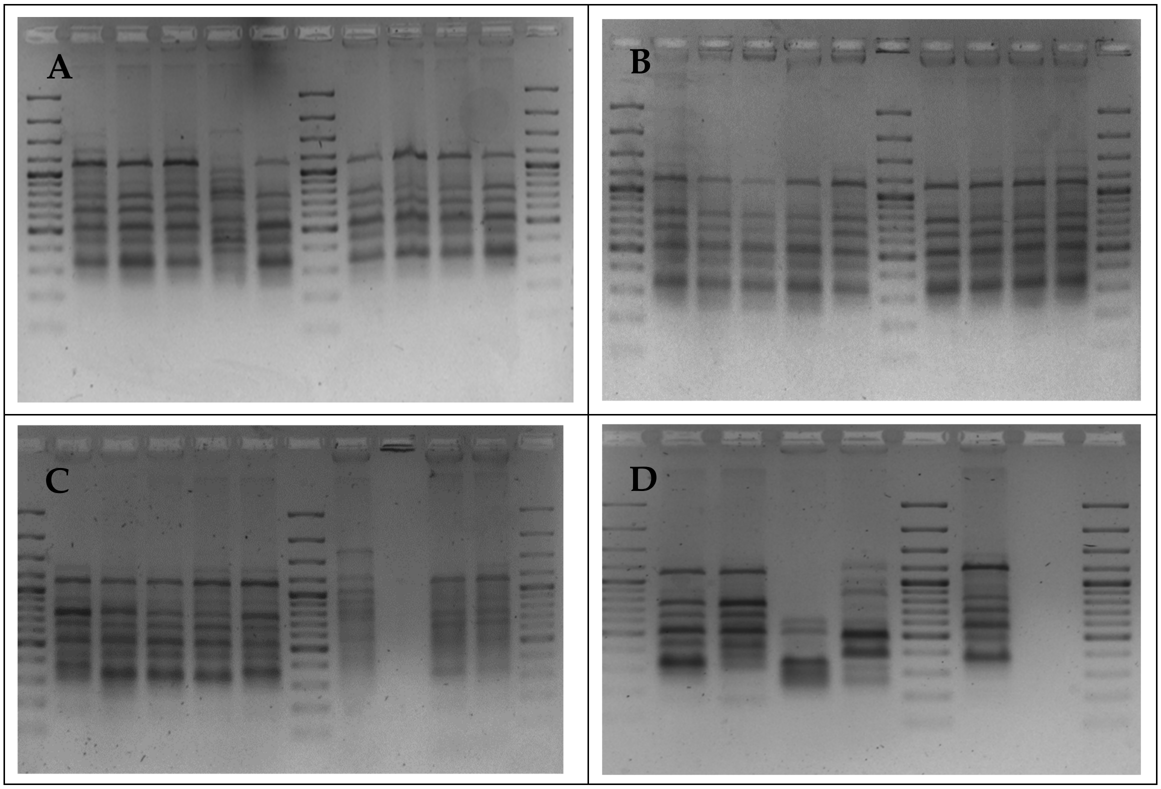

Figure S1. MP-PCR HindIII profiles of *Staphylococcus* genomes. Panel A: 1, 7, 12 – marker 100bp, 2-6, 8-11 – S. aureus_058PP2016-066PP2016; Panel B: 1, 7, 12 – marker 100bp, 2-6, 8-11 – S. aureus_067PP2016-075PP2016; Panel C: 1, 7, 12 – marker 100bp, 2-6, 8-11 – S. aureus_076PP2016-084PP2016; Panel D: 1, 6, 9 – marker 100bp, 2-5, 7 – S. aureus_089PP2016-090PP2016, S. CNS_002PP2016-003PP2016, S. aureus_091PP2016.

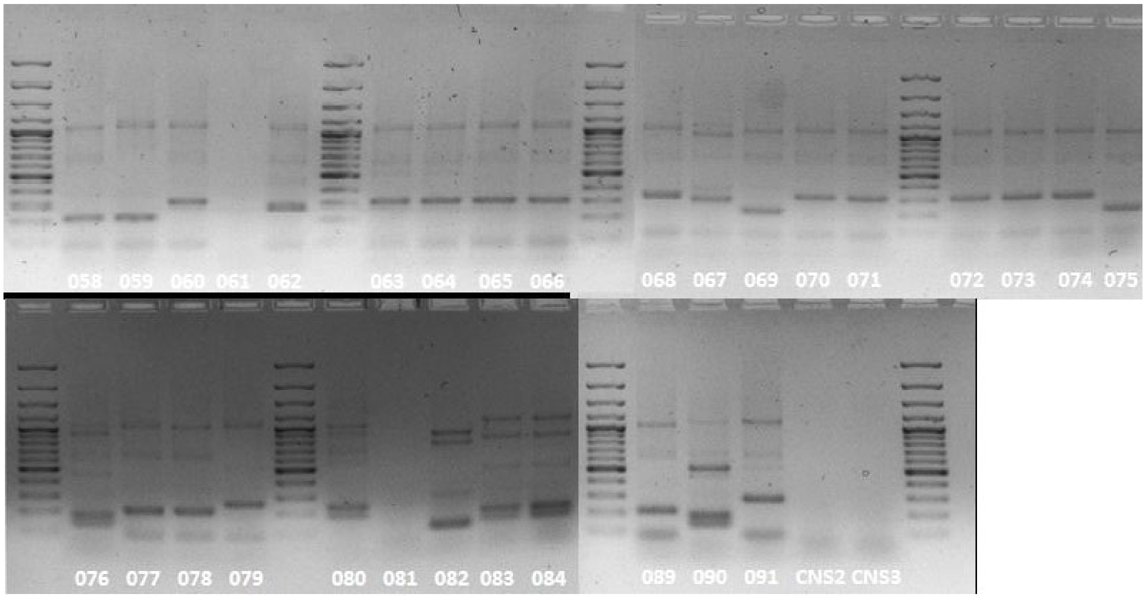

Figure S2. MLVF-PCR_profiles of *Staphylococcus* genomes: marker 100bp, S. aureus_058PP2016-084PP2016, S. aureus_089PP2016-091PP2016, S. CNS_002PP201.
